# Psychosocial Predictors of Anxiety and Depression in Community-Dwelling Older Adults During a Prolonged Infectious Disease Crisis

**DOI:** 10.3390/healthcare14010048

**Published:** 2025-12-24

**Authors:** Nam Hee Kim, Seung Hyun Hong, Hyun Jae Park, Sung Hee Shin

**Affiliations:** 1 Department of Nursing, Graduate School, Kyung Hee University, Seoul 02447, Republic of Korea; nhk0916@korea.kr (N.H.K.); hshsky@khu.ac.kr (S.H.H.); starzzz93@khu.ac.kr (H.J.P.); 2 College of Nursing Science, Kyung Hee University, Seoul 02447, Republic of Korea

**Keywords:** older adults, anxiety, depression, infectious disease-related stress, social support, community-based intervention, mental health

## Abstract

**Highlights:**

**What are the main findings?**

- Infectious-disease-related stress among older adults is multidimensional, and difficulties in social distancing—rather than fear of infection—were the most influential predictor of anxiety and depression.
- Emotional support was the strongest protective factor for both anxiety and depression, while material support provided extra protection specifically for depression.

**What are the implications of the main findings?**

- Mental health interventions should focus on maintaining social connectedness and reducing distancing-related strain, rather than emphasizing infection fear alone.
- Community-based emotional support systems and reliable resource delivery networks should be integrated into routine public health preparedness to sustain older adults’ well-being during prolonged crises.

**Abstract:**

Background/Objectives: Infectious disease outbreaks are recurring global crises that particularly impact older adults, who are vulnerable both biologically and psychosocially. Older adults living in the community, often depending on informal support rather than institutional care, may be especially at risk during extended outbreaks. This study examined psychosocial predictors of anxiety and depression with a focus on the novel integration of multidimensional infectious-disease-related stress and differentiated functional pathways of social support. Methods: A cross-sectional survey involved 178 community-dwelling adults aged 65 and older in South Korea. Validated tools measured anxiety (K-GAI), depression (K-GDS-SF), infectious disease-related stress (fear of infection, anger toward others, and social distancing difficulties), social support (emotional, informational, material, and appraisal), and chronic illness status. Data analysis included correlation analyses and stepwise multiple regression. Results: Difficulties adhering to social distancing were the strongest stress-related predictor of both anxiety and depression, while emotional support emerged as the most powerful protective factor against both outcomes. Material support uniquely mitigated depressive symptoms, and older adults with chronic illness showed heightened vulnerability to depression. Conclusions: Infectious-disease-related stress is multidimensional, extending beyond fear of infection to include social-participation disruption and relational strain. Findings highlight that different types of social support exert distinct protective effects through function-specific mechanisms, reinforcing the importance of targeted intervention design. Practical implications include strengthening emotional-support infrastructure, implementing hybrid digital–offline outreach models, and prioritizing resource allocation for medically vulnerable older adults as part of preparedness planning for future prolonged public-health emergencies.

## 1. Introduction

Inevitable public health crises are becoming recurring realities rather than isolated events, and they are associated with substantial mental health burdens including depression, anxiety [1,2]. Experiences with COVID-19, influenza, and earlier outbreaks such as SARS and MERS demonstrate both the rising frequency of such events and their disproportionate effects on vulnerable groups. Older adults bear a double burden: increased biological vulnerability to infection and accumulated psychosocial stress from isolation, uncertainty, and social network disruptions. Those living in the community, especially, face greater risks due to limited institutional protection and often have reduced access to healthcare, mobility, and informal support [3,4,5]. These recurring emergencies highlight the importance of viewing infectious disease crises not just as biomedical issues but also as ongoing social disasters that demand continuous psychosocial attention and intervention. Despite this acknowledgment, much of the existing literature has viewed infectious-disease-related stress mainly as fear of infection or uncertainty [6,7,8]. However, prolonged public health emergencies produce a wider range of stressors, including frustration with ongoing social distancing, anger toward those perceived as violating health norms, feelings of helplessness due to prolonged restrictions, and secondary stressors such as disrupted routines and limited access to resources [9,10]. Consistent with disaster mental health research perspectives [9], infectious-disease-related stress should be seen as a complex phenomenon that goes beyond just fear. Nevertheless, research on this multidimensional stress model among older adults is limited, despite their increased vulnerability to cumulative psychosocial stress [11]. Recent studies also highlight that social isolation, loneliness, and disrupted social connectedness—not fear of infection alone—are primary predictors of psychological distress among older adults during pandemics [12,13]. Social and technological inequalities increase older adults’ vulnerability. Unlike younger groups who maintain social ties through digital means, older adults often face both digital and social exclusion, which worsens loneliness, isolation, anxiety, and depression [14,15,16,17,18]. Prolonged crises can also lead to stigma, discrimination, and intergenerational conflicts, reducing resilience and lowering quality of life [9,19,20]. From a neurobiological perspective, stigma and chronic social threat activate the hypothalamic–pituitary–adrenal (HPA) axis and disrupt prefrontal–amygdala regulation, increasing emotional reactivity and vulnerability to anxiety and depression in later life [21,22,23,24]. These trends align with evidence that disaster-related stress in later life is connected not only to higher levels of anxiety and depression but also to physical health decline, reduced coping abilities, and increased risks of death and suicide [21,22,23,24,25,26]. Cultural factors may shape these psychological experiences, particularly in highly interdependent societies such as Korea, where collective norms and social expectations influence perceptions of support and exclusion [27].

Social support has long been seen as an essential protective factor during crises [28,29]. However, research often treats it as a single concept, ignoring differences among emotional, informational, material, and appraisal support. According to the buffering hypothesis [28], different types of support may have unique effects. Social support functions as a coping and stress-buffering mechanism, reducing the impact of stress exposure and lowering the likelihood of adverse mental-health outcomes, rather than simply reflecting the quantity of social interaction [16,28]. For older adults, the type and quality of support usually matter more than the amount, especially during extended periods of isolation [16,30]. Mental health is understood here as emotional, psychological, and social well-being that enables individuals to cope with stress, maintain daily functioning, and participate meaningfully in society. While prior research has demonstrated benefits of psychosocial and neurobehavioral approaches (e.g., CBT, neuromodulation), empirical evidence remains limited regarding the differential effectiveness of distinct forms of social support during prolonged infectious-disease outbreaks, especially for community-dwelling older adults [23].

Community-based interventions refer to coordinated, locally delivered programs and services designed to support vulnerable groups through community networks, social structures, and public health resources. This study aimed to explore psychosocial factors predicting anxiety and depression among older adults living in the community during a prolonged infectious disease crisis. It focused on three areas of infectious disease-related stress: fear of infection, anger toward others, and social distancing difficulties—and four types of social support: emotional, informational, material, and appraisal. The study also considered chronic illness status as a vulnerability factor. To our knowledge, it is one of the first to examine simultaneously both the multidimensional stress caused by infectious diseases and the protective roles of different social supports in older adults. Addressing these aspects provides valuable, timely insights to guide targeted interventions and enhance community readiness for future infectious disease emergencies resurgences. This framework offers a conceptual foundation for community-based public health programs by aligning functional mechanisms of social support with context-specific vulnerabilities, ultimately strengthening phase-specific preparedness planning and scalable mental-health protection strategies for older adults.

## 2. Materials and Methods

### 2.1. Research Design

**Figure 1:**
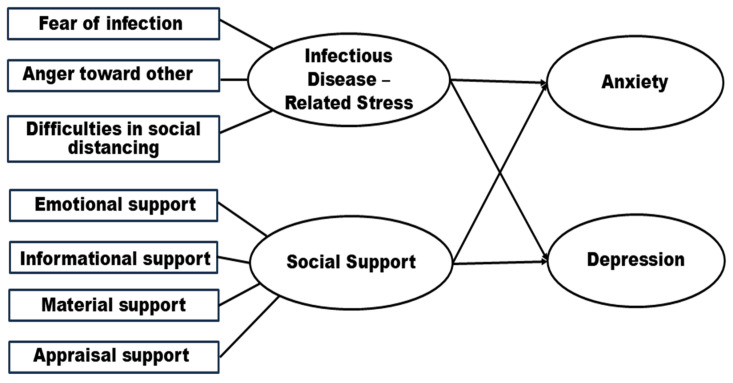
Conceptual framework of the study. This diagram illustrates the hypothesized relationships between multidimensional infectious disease-related stress (such as fear of infection, anger toward others, and social distancing difficulties) and various types of social support (emotional, informational, material, and appraisal) in predicting anxiety and depression among older adults living in the community. The figure was adapted from established theoretical frameworks in the literature and refined by the authors to reflect the objectives of the present study.

This study used a cross-sectional descriptive survey to explore the relationships between stress related to infectious diseases, social support, and mental health outcomes—specifically anxiety and depression—in community-dwelling older adults. As a cross-sectional design, this approach captures data at a single time point and therefore limits causal inference and the ability to examine temporal changes or directional effects. The conceptual framework (Figure 1) depicted infectious-disease-related stress (such as fear of infection, anger toward others, and social distancing challenges) along with four types of social support (e.g., emotional, informational, material, and appraisal) as predictors of anxiety and depression, providing theoretical grounding for the model structure and guiding the selection of analytic strategies. Figure 1 was developed by the authors to illustrate the hypothesized pathways among key variables and to ensure conceptual alignment between research questions and statistical procedures. Stepwise multiple regression was employed to identify the most influential predictors among several interrelated psychosocial factors. This analytic strategy was selected not only for its exploratory suitability but also based on theoretical perspectives suggesting that different dimensions of social support and infectious-disease-related stress contribute uniquely to mental health outcomes in later life. Although alternative approaches such as hierarchical regression or penalized regression methods (e.g., LASSO) could also be considered to enhance model comparison and robustness, stepwise selection was appropriate for refining predictor sets within an early-stage conceptual framework and for generating hypotheses to guide future confirmatory studies.

### 2.2. Participants

A total of 178 community-dwelling older adults aged 65 years or older participated in the study. Participants were approached within their residential communities through on-site announcements and direct invitations at apartment complexes, senior centers, and neighborhood community facilities in Seoul and Gyeonggi Province. Individuals who were able to communicate independently and voluntarily agreed to participate after receiving study information were eligible. Survey data were collected using either self-administered questionnaires or interviewer-assisted formats, depending on literacy level and participant preference, in order to ensure accessibility. Exclusion criteria included difficulty communicating effectively due to functional or cognitive limitations, inability to complete the questionnaire independently or with assistance, and current residence in hospitals or long-term care facilities. Although no formal cognitive screening tools (e.g., MMSE) were administered, participant comprehension and decision-making capacity were assessed informally using orientation-based questions and observational judgment during the initial interaction to confirm reliability. Procedures to identify and manage psychological distress were available, including supportive listening and referral information for mental-health services if needed.

Participants were recruited between September and October 2021, during ongoing COVID-19 social distancing restrictions. Among 180 individuals approached, 178 valid responses (response rate = 98.9%). The required sample size for multiple regression analysis was estimated using G*Power 3.1.9. (Heinrich Heine University Düsseldorf, Düsseldorf, Germany) assuming a medium effect size (f^2^ = 0.15), α = 0.05, and statistical power of 0.95, indicating that at least 160 participants were required; the final sample exceeded this threshold. A convenience sampling strategy was employed across multiple residential community settings to increase sample heterogeneity; however, full representativeness of the broader older-adult population cannot be assumed, and results should be interpreted with this limitation in mind. All participants provided written informed consent, and confidentiality was maintained through the use of temporary identification codes without collecting personal identifiers.

### 2.3. Instruments

A structured self-report questionnaire was administered, comprising 87 items: 14 on general characteristics, 21 on infectious disease-related stress, 17 on social support, 20 on anxiety, and 15 on depression. Permission to use the tool was secured from the original developers and translators. Validated Korean versions of each measure were selected to ensure linguistic and cultural suitability for older adults.

#### 2.3.1. Infectious Disease-Related Stress

The Korean COVID-19 Stress Scale (K-CSS), developed by Taylor et al. [31] and validated for Korean older adults [32], was used to assess Infectious Disease-Related Stress. This scale comprises 21 items across three domains: fear of infection (9 items), anger toward others (6 items), and social distancing challenges (6 items). Participants rated each item on a 4-point Likert scale (1–4), yielding total scores ranging from 21 to 84. Higher scores indicate higher stress levels. Cronbach’s α for this study was 0.88. Example items include “I am worried about catching the virus” (fear of infection), “I feel angry at people who do not follow quarantine rules” (anger toward others), and “It is difficult to maintain relationships because of distancing restrictions” (social-distancing difficulties). In this study, infectious-disease-related stress specifically refers to stress associated with the COVID-19 pandemic and does not include stress related to non-communicable conditions.

#### 2.3.2. Social Support

Social support was measured using the Korean version of the Multidimensional Scale of Perceived Social Support (K-MSPSS) [33], validated for Korean older adults [34]. This scale comprises 17 items across four subdomains: emotional, informational, material, and appraisal support. Respondents rate each item on a 5-point Likert scale (1–5), yielding total scores ranging from 17 to 85. Higher scores reflect greater perceived support. Cronbach’s α was 0.92. Sample items include “There is someone who understands my feelings” (emotional), “I can receive useful advice when needed” (informational), “Someone helps me when I need physical assistance” (material), and “There is someone who reassures me when I doubt myself” (appraisal). This tool captures the perceived availability and adequacy of functional support from meaningful individuals rather than simply the quantity of social interactions.

#### 2.3.3. Anxiety

Anxiety was assessed using the Korean Geriatric Anxiety Inventory (K-GAI), created by Pachana et al. [35] and validated in Korea by Kim et al. [36]. The tool consists of 20 yes/no items, with total scores ranging from 0 to 20. A score of 7 or higher suggests clinically significant anxiety. Cronbach’s α was 0.94.

#### 2.3.4. Depression

Depression was evaluated using the Korean Geriatric Depression Scale–Short Form (K-GDS-SF), created by Yesavage et al. [37] and validated in Korea by Gi [38]. This instrument consists of 15 yes/no items, yielding a total score of 0 to 15. A score of 5 or higher suggests the presence of depressive symptoms. Cronbach’s α was 0.97.

The clinical cutoff scores applied for both the K-GAI (≥7) and the K-GDS-SF (≥5) were based on validation studies conducted with Korean older adults [36,38], demonstrating high sensitivity and specificity for identifying clinically meaningful anxiety and depressive symptoms in this population. Because psychological symptom thresholds may differ across cultural and linguistic contexts, the use of Korean-validated cutoff criteria enhances the cultural appropriateness and interpretive accuracy of the findings.

### 2.4. Data Collection and Ethical Considerations

Before data collection, approval was secured from the Institutional Review Board of Kyung Hee University in Seoul, South Korea [KHSIRB-21-346(RA)]. The study adhered to the ethical principles outlined in the Declaration of Helsinki. Data were collected in September 2021, about 18 months after the start of the COVID-19 pandemic, when social distancing and mobility restrictions were still in effect. Participants were approached within their residential communities, such as apartment complexes, neighborhood gathering spots, and local senior centers, and invited to participate in the survey. Trained research assistants explained the study’s purpose and procedures, and written informed consent was obtained from all participants.

Surveys were administered through self-administered paper questionnaires or interviewer-assisted formats depending on participants’ literacy needs and functional abilities, ensuring accessibility for older adults with diverse capabilities. Standardized procedural guidelines and scripted prompts were used throughout survey administration to enhance consistency and minimize interviewer influence. A structured protocol outlining administration procedures was developed to support interrater consistency should additional data collectors be needed in future phases. To evaluate potential selection bias, response and refusal rates were recorded: 180 individuals were approached, of whom 178 agreed to participate and 2 declined due to personal or time constraints (response rate = 98.9%). Although high, this response rate does not eliminate the possibility of self-selection bias, which should be considered when interpreting findings.

### 2.5. Data Analysis

The data were processed using IBM SPSS Statistics for Windows, version 25.0 (IBM Corp., Armonk, NY, USA). Participants’ demographics were summarized with frequencies, percentages, means, and standard deviations. The levels of infectious-disease-related stress, social support, anxiety, and depression were assessed through means, standard deviations, skewness, and kurtosis. Absolute values of skewness and kurtosis below 2.0 were considered to demonstrate a normal distribution [39]. Differences in anxiety and depression according to participants’ demographics were analyzed using independent t-tests and one-way ANOVAs, with Scheffé’s test applied for *post hoc* comparisons. Pearson’s correlation coefficients were computed to examine bivariate relationships among infectious-disease-related stress, social support, anxiety, and depression. Stepwise multiple regression analysis was conducted to identify psychosocial predictors of anxiety and depression. This procedure was selected given the study’s exploratory aim and to avoid multicollinearity, as tolerance values and variance inflation factors (VIFs) confirmed the absence of multicollinearity (tolerance > 0.1; VIF < 10). Reliability for each instrument was assessed using Cronbach’s α.

## 3. Results

### 3.1. General Characteristics and Group Differences in Anxiety and Depression

This study included 178 older adults living in the community, with an average age of 74.1 years. Among participants, 48.3% were aged 65–69, 38.8% were aged 70–79, and 12.9% were 80 or older. Women comprised 57.3% of the sample and men 42.7%. most participants were married, lived with others, and had at least one chronic illness. Regarding education, approximately 40% had completed middle school or less, and most participants were covered by the national health insurance program, with a smaller proportion receiving medical aid (Table 1). Group comparisons showed that anxiety and depression were significantly higher among participants with lower education levels, those receiving medical aid, and those rating their health as poor. Depression was also higher among those without religion, with chronic conditions, and with fewer supportive members (Table 1). Overall, the observed group differences in anxiety and depression were of small to moderate magnitude (η^2^ = 0.06–0.13), suggesting that although statistically significant, the practical impact of individual sociodemographic factors was modest.

### 3.2. Levels of Anxiety, Depression, Infectious Disease-Related Stress, and Social Support

Descriptive statistics for key study variables are presented in Table 2. The average anxiety score was 5.87 (SD = 6.08), and the average depression score was 5.99 (SD = 4.47). The mean total score for infectious-disease-related stress was 57.40 (SD = 12.11). Among its subdomains, fear of infection had the highest average (M = 24.28, SD = 6.74), followed by anger toward others (M = 18.69, SD = 4.15) and difficulties in social distancing (M = 14.43, SD = 4.70). The overall social support score averaged 58.99 (SD = 15.41). Of the four support domains, emotional support had the highest mean (M = 28.46, SD = 7.59), while material support was the lowest (M = 9.74, SD = 3.35). Informational support (M = 10.29, SD = 2.98) and appraisal support (M = 10.51, SD = 3.04) scored at intermediate levels.

### 3.3. Correlations Among Anxiety, Depression, Infectious Disease-Related Stress, and Social Support

Table 3 presents the correlations among anxiety, depression, infectious disease-related stress, and social support. Anxiety was moderately positively associated with overall infectious-disease-related stress (r = 0.48, *p* < 0.001) and moderately negatively associated with social support (r = −0.46, *p* < 0.001). Similarly, depression showed a moderate positive association with infectious-disease-related stress (r = 0.52, *p* < 0.001) and a moderately strong negative association with social support (r = −0.58, *p* < 0.001). These patterns indicate that higher stress related to the pandemic is linked to greater psychological distress, whereas higher perceived social support is linked to lower anxiety and depression. Among the subdomains of infectious-disease-related stress, difficulties in social distancing showed significant positive correlations with both anxiety (r = 0.30, *p* < 0.001) and depression (r = 0.24, *p* = 0.002), highlighting that challenges in maintaining relationships and routines under prolonged distancing may be particularly important for older adults’ mental health. In contrast, fear of infection and anger toward others were not significantly related to anxiety or depression, suggesting that not all facets of pandemic-related stress translate directly into internalizing symptoms.

**Figure 2:**
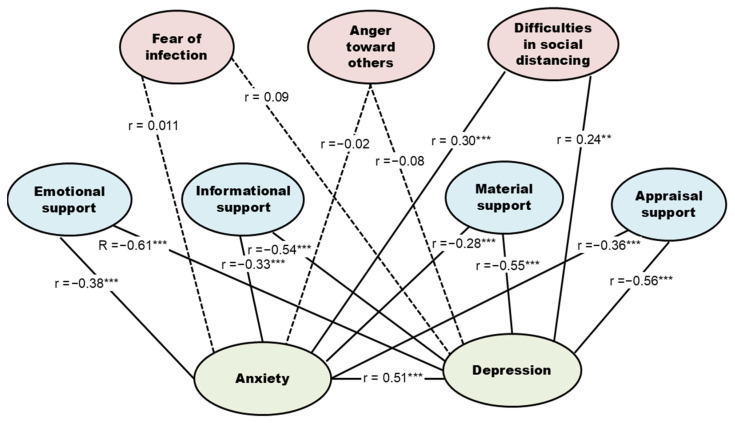
Correlation model of anxiety, depression, infectious disease-related stress, and social support in community-dwelling older adults. Solid lines represent significant correlations, while dashed lines indicate nonsignificant ones. Values represent Pearson’s *r*. Statistical significance: *** *p* < 0.001, ** *p* < 0.01.

All four subdomains of social support were significantly negatively correlated with both anxiety and depression, with emotional support showing the strongest negative correlation (*r* = −0.38, *p* < 0.001 for anxiety; *r* = −0.61, *p* < 0.001 for depression). Similarly, appraisal (*r* = −0.36, *p* < 0.001; *r* = −0.56, *p* < 0.001), informational (*r* = −0.33, *p* < 0.001; *r* = −0.54, *p* < 0.001), and material support (*r* = −0.28, *p* < 0.001; *r* = −0.55, *p* < 0.001) all had significant negative associations. These patterns are depicted visually in Figure 2, which shows the relationships among infectious disease-related stress dimensions, social support subdomains, and the outcomes of anxiety and depression. These relationships are summarized in Figure 2, which visually distinguishes significant and non-significant correlations using solid and dashed lines and highlights emotional support and social-distancing difficulties as central correlates of anxiety and depression.

### 3.4. Predictors of Anxiety and Depression in Community-Dwelling Older Adults

Stepwise multiple regression analyses were performed to determine the predictors of anxiety and depression in community-dwelling older adults. The results are shown in Table 4. For anxiety, the regression model was statistically significant (F = 12.80, *p* < 0.001), with an explanatory power of 22.4% (R^2^ = 0.22, Adjusted R^2^ = 0.22). Two variables were retained in the final model. Emotional support had a significant negative effect (β = −0.37, *p* < 0.001), indicating that greater emotional support was associated with lower anxiety. In contrast, difficulties with social distancing had a significant positive effect (β = 0.29, *p* < 0.001), suggesting that higher stress from prolonged distancing was associated with greater anxiety. The Durbin–Watson statistic was 2.01, indicating no autocorrelation. All regression assumptions were met, with tolerance values above 0.1 and VIFs below 10.

For depression, the final model was statistically significant (F = 26.40, *p* < 0.001), explaining 44.7% of the variance (R^2^ = 0.45, Adjusted R^2^ = 0.46). Four predictors remained in the model. Emotional support (β = −0.38, *p* < 0.001) and material support (β = −0.25, *p* < 0.001) showed significant negative effects, confirming their protective role in reducing depressive symptoms. In contrast, difficulties in social distancing (β = 0.22, *p* < 0.001) and the presence of chronic illness (β = 0.15, *p* = 0.009) had significant positive effects, indicating higher vulnerability to depression. The Durbin–Watson statistic was 2.10, and diagnostic tests confirmed the absence of multicollinearity and satisfaction of regression assumptions.

**Figure 3:**
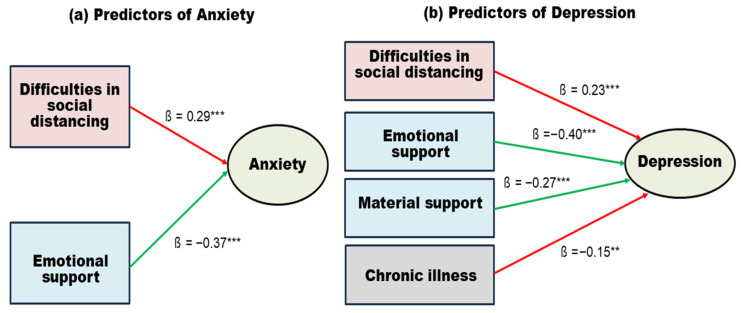
Predictors of anxiety (**a**) and depression (**b**) among community-dwelling older adults are displayed. Standardized regression coefficients (β) are shown along the arrows. Red arrows indicate positive associations, representing stress-related risk factors, while green arrows indicate negative associations, reflecting the protective effects of social support. Statistical significance: *** *p* < 0.001, ** *p* < 0.01.

These results indicate that higher emotional and material support are associated with fewer depressive symptoms, whereas greater difficulties in social distancing and having a chronic illness confer increased risk for depression. The Durbin–Watson statistic was 2.10, and there was no evidence of multicollinearity, indicating that the model satisfied key assumptions. Figure 3 illustrates these findings by depicting emotional and material support as protective factors and social-distancing difficulties and chronic illness as risk factors, visually emphasizing the relative strength of each predictor for anxiety and depression.

## 4. Discussion

This study explored psychosocial factors influencing anxiety and depression in older adults living in the community during an extended infectious disease crisis. The findings highlight the importance of considering a multidimensional approach to infectious-disease-related stress and understanding the specific protective effects of social support. Although demographic factors such as education level and perceived health status contributed to differences in anxiety and depression, effect sizes were small to moderate (η^2^ = 0.06–0.13), indicating that these variables accounted for limited variance. This reinforces the central importance of psychosocial mechanisms—particularly emotional and material support—rather than sociodemographic status alone. Unlike previous studies that examined pandemic-related distress primarily from fear-based perspectives or assessed social support as a unitary construct, this study uniquely integrates a multidimensional infectious-disease-related stress model with differentiated support pathways, offering a more nuanced psychosocial mechanism explaining mental-health vulnerability in later life.

Our findings empirically show that stress related to infectious diseases is not just about fear of infection but involves multiple interconnected areas, such as anger toward others and challenges in social distancing. Among these, struggles with social distancing were the most consistent predictors of anxiety and depression. This underscores that restrictions on social participation and face-to-face contact, more than the fear of infection itself, impose significant psychological stress on older adults. Similar international studies suggest that extended isolation, disrupted routines, and strained social relationships often have a greater impact on mental health in later life than infection concerns alone [3,4,5,39,40]. From a neurobiological perspective, prolonged isolation activates the hypothalamic–pituitary–adrenal (HPA) axis and disrupts prefrontal–amygdala regulation, heightening emotional reactivity and vulnerability to anxiety and depression in later life [21,22,23,24]. Further, chronic stress and social disconnection may impair hippocampal regulation of the HPA axis and reduce serotonergic and dopaminergic signaling, weakening executive emotional control mediated by the prefrontal cortex and increasing amygdala-driven threat responses. Such dysregulation offers a mechanistic explanation for why distancing-related strain demonstrated stronger effects than infection-related fear. Notably, fear of infection and anger toward others were not significant predictors of anxiety or depression in this study. Although statistically non-significant, these stress domains may still hold behavioral and policy relevance by influencing preventive adherence, public-health cooperation, and community cohesion. Future research should explore whether these domains affect mental-health outcomes indirectly through pathways such as perceived risk, collective efficacy, or social conflict, and whether their impact varies across different phases of a crisis or cultural contexts.

Second, the findings indicate that different types of social support do not have the same level of effectiveness. Emotional support emerged as the most potent safeguard against anxiety and depression, whereas material support provided an extra layer of protection specifically against depression. These results support the buffering hypothesis [28] and recent gerontological studies that highlight qualitative aspects of support, such as reassurance, empathy, and tangible help, are more impactful than the number of social contacts [16,33,40]. Recent studies extend these insights, demonstrating that social support protects against depression, anxiety, and loneliness in older adults [41,42], and operates through mediating pathways involving loneliness, economic resources, and health behaviors [43,44]. Additional evidence shows that social support and self-efficacy enhance hopefulness [45], and that social capital and network building play crucial roles in improving quality of life during crises [44,46]. These insights help improve intervention strategies by highlighting the need for tailored function-specific support methods to address various mental health concerns and outcomes effectively. Collectively, these findings emphasize the need for support frameworks that are functionally matched to specific psychological needs, rather than generalized or volume-based approaches. Importantly, the differential effects observed in this study—where emotional support protected against both anxiety and depression, whereas material support uniquely mitigated depressive symptoms—suggest that emotional reassurance may buffer acute psychological distress, while tangible assistance may help counter resource-related burdens that accumulate over time. In addition, effect sizes for key predictors suggest substantive clinical relevance, indicating that even modest improvements in emotional support or reductions in social-distancing strain may yield meaningful mental-health benefits at the population level. This distinction underscores the importance of designing multidimensional support frameworks that match the functional purpose of each type of support rather than applying generalized or uniform approaches for older adults during prolonged infectious disease crises.

Third, chronic illness was identified as an independent predictor of depression, emphasizing the increased vulnerability of older adults who confront both physical and psychological challenges. This finding matches WHO reports and recent epidemiological data showing that comorbid conditions heighten susceptibility to depression, anxiety, and reduced resilience during infectious disease crises [1,2,41]. Neurobiological research suggests that chronic inflammation associated with long-term illness may exacerbate stress reactivity and impair neural pathways regulating mood, increasing vulnerability to depression under prolonged crisis conditions [13]. These findings emphasize the importance of targeted psychosocial and medical support for older adults with chronic diseases within disaster-preparedness frameworks. These results signal the need to incorporate multimorbidity-sensitive risk stratification systems within aging services and emergency preparedness frameworks, ensuring that resource allocation is prioritized based on cumulative psychosocial and medical vulnerability.

From a practice perspective, phase-specific intervention strategies are needed. From an intervention standpoint, our findings suggest that strategies should be phase-specific. During preparedness, strengthening emotional support networks, enhancing digital accessibility, and establishing delivery systems for essential goods can bolster resilience. In the acute response phase, interventions such as psychological first aid, support hotlines, and prioritizing vulnerable groups (e.g., individuals with less formal education, medical aid recipients) are crucial. During prolonged restriction phases, hybrid digital–offline community programs can alleviate distancing-related distress and reduce digital-access disparities [47]. In recovery stages, restoring social participation and screening for depression and cognitive decline remain priorities, recognizing that pandemic experiences may produce long-term consequences [5,19,27]. Additionally, future resilience-oriented models should incorporate adaptive intervention thresholds based on dynamic risk profiling, enabling personalized escalation of psychosocial support when early indicators—such as declining emotional support or increasing distancing-related strain—are detected. Such precision-oriented frameworks may enhance the timeliness and effectiveness of mental-health responses during prolonged crises.

Policy implications of these findings are also notable. During COVID-19, the Korean government introduced initiatives such as mental health support call programs and emergency supply distribution, offering timely emotional and material support to older adults [48]. Institutionalizing such programs within routine public health systems could embed protective mechanisms for future crises [48,49,50]. Telecare interventions have demonstrated effectiveness in reducing depression and anxiety and improving quality of life [51], reinforcing the value of integrating digital platforms and hybrid outreach into community-based preparedness systems. This study’s academic contribution lies in explicitly modeling the multidimensional nature of pandemic-related stress and differentiating the effects of distinct forms of social support, highlighting the importance of integrating neurobiological vulnerability pathways with psychosocial mechanisms. It also highlights the importance of integrating neurobiological vulnerability pathways with psychosocial mechanisms to better understand mental-health outcomes in later life. Furthermore, emerging technologies such as Ecological Momentary Assessment (EMA) and Just-In-Time Adaptive Interventions (JITAIs) offer promising opportunities for real-time monitoring and personalized mental-health support [46,51].

Nevertheless, several limitations should be recognized. First, cross-sectional design limits causal interpretation, and the use of convenience sampling from limited urban and peri-urban regions may restrict generalizability to rural or institutionalized populations. Second, reliance on self-report questionnaires introduces the possibility of recall and social-desirability bias, and the potential for common-method variance should also be considered. Third, although descriptive subgroup comparisons of age and education were examined, no clear interaction pattern was observed, and formal interaction testing was not included; future research should investigate these differences more systematically. Fourth, formal cognitive screening (e.g., MMSE) was not conducted, although interviewers confirmed participants’ ability to understand survey content. Fifth, detailed socioeconomic indicators such as income or housing type were not collected, despite their known effects on social support and mental-health outcomes. Future studies should incorporate standardized cognitive assessments, longitudinal and cross-cultural samples, and more comprehensive socioeconomic measures to improve external validity. Additionally, cultural and contextual characteristics specific to Korean community structures—such as strong collectivism, family-centered support networks, and community-based decision-making norms—may influence perceptions of social support and stress responses, potentially limiting generalizability to more individualistic cultural settings. Future studies should examine cross-cultural variations to better understand sociocultural mechanisms underlying mental-health vulnerability among older adults.

In summary, this study demonstrates that diverse stressors and differentiated types of social support significantly influence mental-health outcomes among community-dwelling older adults during infectious-disease crises. By integrating psychosocial perspectives with neurobiological vulnerability processes, these findings highlight the need for targeted, function-specific interventions to support mental health in aging populations. As infectious-disease emergencies are likely to recur, tailored interventions—from emotional reassurance to digital-hybrid service delivery—are essential to enhance resilience and protect psychological well-being in later life. Collectively, these results underscore that addressing social-disconnection stressors and expanding emotional-support infrastructures may yield substantial public-health impact, especially when embedded within scalable digital–community hybrid systems capable of early detection and adaptive intervention.

## 5. Conclusions

This study showed that stress related to infectious diseases in community-dwelling older adults is multidimensional, including fear of infection, anger toward others, and challenges in social distancing [31,32]. Among these stressors, difficulties in social distancing emerged as the strongest predictor of anxiety and depression [5,12,20], emphasizing that psychosocial stressors during prolonged crises can be as consequential as biological vulnerability [1,2]. Social support showed functionally distinct effects: emotional support served as the most robust protective factor for both anxiety and depression [16,17], while material support additionally reduced depressive symptoms [44], highlighting the need for targeted, quality-focused rather than generalized support approaches. Given the effect sizes observed, even incremental improvements in emotional support or reductions in distancing-related strain may yield meaningful population-level benefits [12,30], reinforcing the importance of proactive intervention strategies. In practical terms, the findings suggest the need to strengthen community-based emotional support networks, expand hybrid digital–offline participation opportunities, and improve access to digital resources for socially isolated older adults [45,47]. Public-health systems may also benefit from integrating sustainable telecare services and coordinated outreach mechanisms [41,49] to support older adults during prolonged infectious-disease crises.

In conclusion, applying a precision public-health perspective that aligns multidimensional stress assessment with differentiated support pathways may offer a conceptual foundation for scalable and equity-focused mental-health strategies capable of responding to recurrent crises [49,52]. Future research should employ longitudinal and cross-cultural designs and explore technology-supported intervention models such as EMA and JITAIs to enable timely, personalized support for aging populations [50].

## Figures and Tables

**Table 1:** General characteristics of participants and differences in anxiety and depression (*n* = 178).

Variables	Categories	*n* (%)	Anxiety			Depression		
			Mean ± SD	t or F (*p*)	η^2^	Mean ± SD	t or F (*p*)	η^2^
Sex	Male	76 (42.7)	5.89 ± 6.76	0.04 (0.965)		5.75 ± 4.70	−0.61 (0.540)	
	Female	102 (57.3)	5.85 ± 5.56			6.17 ± 4.31		
Age	65~69	86 (48.3)	5.57 ± 5.70	0.31 (0.737)		5.55 ± 4.44	0.82 (0.441)	
	70~79	69 (38.8)	5.99 ± 6.72			6.36 ± 4.69		
	≥80~	23 (12.9)	6.65 ± 5.56			6.52 ± 3.92		
Marital Status	Widow/Widower	42 (23.6)	7.21 ± 6.23	3.25 (0.156)		7.29 ± 4.50	2.53 (0.082)	
	Married	125 (70.2)	5.16 ± 5.82			5.52 ± 4.38		
	Unmarried	11 (6.2)	8.82 ± 7.14			6.36 ± 4.90		
Living situations	Alone	39 (21.9)	5.70 ± 5.98	−0.72 (0.475)		5.87 ± 4.33	−0.67 (0.507)	
	Lives with another	139 (78.1)	6.49 ± 6.48			6.41 ± 4.98		
Religion	Yes	121 (68.0)	5.81 ± 5.97	−0.19 (0.846)		5.52 ± 4.54	−2.05 (0.042)	
	No	57 (32.0)	6.00 ± 6.36			6.98 ± 4.19		
Education level	≤Middle ^a^	72 (40.5)	7.88 ± 6.54	6.95 (0.001) ^a > b,c †^	0.07	7.51 ± 4.34	8.60 (<0.001) ^a > b,c †^	0.09
	High ^b^	57 (32.0)	4.86 ± 5.65			5.49 ± 4.58		
	≥College ^c^	49 (27.5)	4.10 ± 5.05			4.33 ± 3.87		
Medical Insurance type	National basic living security	30 (16.9)	9.93 ± 6.90	3.64 (0.001)		9.33 ± 4.09	4.76 (<0.001)	
	General Health insurance	148 (83.1)	5.05 ± 5.57			5.31 ± 4.25		
Perceived health status	Bad ^a^	41 (23.0)	8.51 ± 6.13	6.02 (0.003) ^a > b,c †^	0.06	9.44 ± 3.69	33.14 (<0.001) ^a > b > c †^	0.28
	Moderate ^b^	74 (41.6)	5.64 ± 6.01			6.04 ± 4.56		
	Good ^c^	63 (35.4)	4.43 ± 5.66			3.68 ± 3.26		
Disease	Yes	134 (75.3)	5.96 ± 6.07	0.35 (0.726)		6.37 ± 4.58	2.02 (0.045)	
	No	44 (24.7)	5.59 ± 6.18			4.82 ± 3.94		
Activity place	Yes	141 (79.2)	6.19 ± 6.22	1.38 (0.170)		6.05 ± 4.65	0.40 (0.693)	
	No	37 (20.8)	4.65 ± 5.44			5.76 ± 3.80		
Number of supportive members	0~2 ^a^	51 (28.7)	7.71 ± 6.06	3.40 (0.060)		8.37 ± 4.12	12.81 (<0.001) ^a > b,c †^	0.13
	3~4 ^b^	58 (32.6)	4.95 ± 5.92			5.69 ± 4.39		
	≥5 ^c^	69 (38.7)	5.29 ± 6.02			4.48 ± 4.11		
COVID-19 experience	Yes	7 (3.9)	5.71 ± 6.50	−0.07 (0.945)		7.14 ± 5.52	0.70 (0.488)	
	No	171 (96.1)	5.88 ± 6.08			5.94 ± 4.44		
Vaccination	Yes	174 (97.8)	5.88 ± 6.12	0.12 (0.902)		5.90 ± 4.47	−1.83 (0.070)	
	No	4 (2.2)	5.50 ± 4.43			10.00 ± 2.16		

Multiple response; ^†^ Scheffé; ^a–c^: Group labels used for post hoc comparisons; SD = Standard Deviation.

**Table 2:** Level of Infectious Disease-Related Stress, and Social Support, Anxiety, Depression (*n* = 178).

Variables	Mean	SD	Min	Max	Skewness	Kurtosis
Infectious Disease-Related Stress	57.40	12.11	5	84	−0.79	1.57
Fear of infection	24.28	6.74	3	36	−0.78	0.58
Anger toward others	18.69	4.15	0	24	−1.16	2.47
Difficulties in social distancing	14.43	4.70	1	24	−0.40	0.00
Social Support	58.99	15.41	17	85	−0.47	−0.01
Emotional support	28.46	7.59	8	40	−0.59	0.06
Informational support	10.29	2.98	3	15	−0.46	−0.16
Material support	9.74	3.35	3	15	−0.12	−0.90
Appraisal support	10.51	3.04	3	15	−0.37	−0.37
Anxiety	5.87	6.08	0	20	0.81	−0.63
Depression	5.99	4.47	0	15	0.36	−1.13

SD = Standard Deviation; Min = Minimum; Max = Maximum.

**Table 3:** Correlations between Anxiety, Depression, Infectious Disease-Related Stress, and Social Support (*n* = 178).

Variables	1.1	1.2	1.3	2.1	2.2	2.3	2.4	3	4
						r(*p*)			
1.1. Fear of infection	1								
1.2. Anger toward others	0.46 (<0.001)	1							
1.3. Difficulties in social distancing	0.39 (<0.001)	0.29 (<0.001)	1						
2.1. Emotional support	−0.02 (0.787)	0.25 (0.001)	−0.03 (0.654)	1					
2.2. Informational support	0.01 (0.922)	0.26 (<0.001)	0.03 (0.670)	0.81 (<0.001)	1				
2.3. Material support	0.02 (0.777)	0.22 (0.003)	−0.04 (0.581)	0.68 (<0.001)	0.70 (<0.001)	1			
2.4. Appraisal support	0.06 (0.429)	0.23 (0.002)	−0.07 (0.346)	0.76 (<0.001)	0.78 (<0.001)	0.81 (<0.001)	1		
3. Anxiety	0.11 (0.165)	−0.02 (0.764)	0.30 (<0.001)	−0.38 (<0.001)	−0.33 (<0.001)	−0.28 (<0.001)	−0.36 (<0.001)	1	
4. Depression	0.09 (0.227)	−0.08 (0.278)	0.24 (0.002)	−0.61 (<0.001)	−0.54 (<0.001)	−0.55 (<0.001)	−0.56 (<0.001)	0.51 (<0.001)	1

**Table 4:** Predictors of Anxiety and Depression by Infectious Disease-Related Stress and Social Support (*n* = 178).

Independent Variables	Dependent Variables	B	S.E.	β	t(*p*)	R^2^	Adjusted R^2^	F(*p*)	Durbin- Watson
Emotional support *		−0.29	0.05	−0.37	−5.48 (<0.001)	0.22	0.22	25.33 (<0.001)	2.01
Difficulties in social distancing *	Anxiety	0.38	0.09	0.29	4.36 (<0.001)				
Emotional support **		−0.24	0.05	−0.40	−5.32 (<0.001)	0.47	0.46	38.18 (<0.001)	2.10
Difficulties in social distancing **	Depression	0.22	0.05	0.23	4.06 (<0.001)				
Material support **		−0.36	0.10	−0.27	−3.57 (<0.001)				
Disease (ref: Yes) **		−1.54	0.58	−0.15	−2.66 (0.009)				

* control variables = education level, medical insurance type, perceived health status ** control variables: religion, education level, medical insurance type, perceived health status, disease, number of supportive peers.

## Data Availability

The data presented in this study is available on request from the corresponding author. The data is not publicly available due to privacy or ethical restrictions.

## Abbreviations

The following abbreviations are used in this manuscript:

COVID-19	Coronavirus Disease 2019
EMA	Ecological Momentary Assessment
JITAI	Just-in-Time Adaptive Intervention
K-CSS	Korean COVID-19 Stress Scale
K-GAI	Korean Geriatric Anxiety Inventory
K-GDS-SF	Korean Geriatric Depression Scale—Short Form
K-MSPSS	Korean version of the Multidimensional Scale of Perceived Social Support
MERS	Middle East Respiratory Syndrome
SARS	Severe Acute Respiratory Syndrome
